# Predicting Risky Sexual Behavior Among College Students Through Machine Learning Approaches: Cross-sectional Analysis of Individual Data From 1264 Universities in 31 Provinces in China

**DOI:** 10.2196/41162

**Published:** 2023-01-25

**Authors:** Xuan Li, Hanxiyue Zhang, Shuangyu Zhao, Kun Tang

**Affiliations:** 1 Vanke School of Public Health Tsinghua University Beijing China

**Keywords:** risky sexual behavior, sexually transmitted infections, college students, machine learning, prediction, students, risk factor, STI, intervention, China, sex

## Abstract

**Background:**

Risky sexual behavior (RSB), the most direct risk factor for sexually transmitted infections (STIs), is common among college students. Thus, identifying relevant risk factors and predicting RSB are important to intervene and prevent RSB among college students.

**Objective:**

We aim to establish a predictive model for RSB among college students to facilitate timely intervention and the prevention of RSB to help limit STI contraction.

**Methods:**

We included a total of 8794 heterosexual Chinese students who self-reported engaging in sexual intercourse from November 2019 to February 2020. We identified RSB among those students and attributed it to 4 dimensions: whether contraception was used, whether the contraceptive method was safe, whether students engaged in casual sex or sex with multiple partners, and integrated RSB (which combined the first 3 dimensions). Overall, 126 predictors were included in this study, including demographic characteristics, daily habits, physical and mental health, relationship status, sexual knowledge, sexual education, sexual attitude, and previous sexual experience. For each type of RSB, we compared 8 machine learning (ML) models: multiple logistic regression (MLR), naive Bayes (BYS), linear discriminant analysis (LDA), random forest (RF), gradient boosting machine (GBM), extreme gradient boosting (XGBoost), deep learning (DL), and the ensemble model. The optimal model for both RSB prediction and risk factor identification was selected based on a set of validation indicators. An MLR model was applied to investigate the association between RSB and identified risk factors through ML methods.

**Results:**

In total, 5328 (60.59%) students were found to have previously engaged in RSB. Among them, 3682 (41.87%) did not use contraception every time they had sexual intercourse, 3602 (40.96%) had previously used an ineffective or unsafe contraceptive method, and 1157 (13.16%) had engaged in casual sex or sex with multiple partners. XGBoost achieved the optimal predictive performance on all 4 types of RSB, with the area under the receiver operator characteristic curve (AUROC) reaching 0.78, 0.72, 0.94, and 0.80 for contraceptive use, safe contraceptive method use, engagement in casual sex or with multiple partners, and integrated RSB, respectively. By ensuring the stability of various validation indicators, the 12 most predictive variables were then selected using XGBoost, including the participants’ relationship status, sexual knowledge, sexual attitude, and previous sexual experience. Through MLR, RSB was found to be significantly associated with less sexual knowledge, more liberal sexual attitudes, single relationship status, and increased sexual experience.

**Conclusions:**

RSB is prevalent among college students. The XGBoost model is an effective approach to predict RSB and identify corresponding risk factors. This study presented an opportunity to promote sexual and reproductive health through ML models, which can help targeted interventions aimed at different subgroups and the precise surveillance and prevention of RSB among college students through risk probability prediction.

## Introduction

Risky sexual behavior (RSB) is defined as sexual activities that are more likely to lead to the risk of sexually transmitted infections (STIs) and unwanted pregnancies [1], including sex without contraceptive use and sex with an ineffective or unsafe contraceptive method. It is a serious issue among college students worldwide, especially in low- and middle-income countries [2,3]. Among 5 types of contraceptive methods under the classification criterion from the World Health Organization (WHO) [4], condoms and hormonal contraceptive methods are regarded as safe and highly effective for adolescents [5]. Studies have shown that consistent condom use is low among college students [6]. In previous research, it was estimated that approximately 40% of students did not use a condom during their last sexual encounter [7]. Casual sex and sex with multiple partners have also been regarded as RSBs [8-10]. Nearly half of the college students had casual sex experience [11,12], and approximately 50.7% of students were sexually active, with 42.3% of students having multiple sexual partners [13]. Thus, to help reduce the influence of RSB, it is important to intervene and prevent it among college students by identifying relevant risk factors and making RSB predictions.

A number of association studies have focused on the risk factors for RSB. A systematic review of 30 papers summarized 11 aspects of risk factors of RSB including sociodemographics, gender roles, substance use, and partner characteristics [14]. It was widely validated that being male [15,16], drinking alcohol [17], experiencing poverty, and experiencing peer pressure [18] are significant risk factors for RSB [19,20]. Mental health has also been linked to RSB, with higher depression resulting in more RSB and STIs [20,21]. In addition, numerous public health and sociological studies have found that romantic relationship status [22,23], sexual knowledge [1], and sexual attitude have a significant influence on RSB [24,25]. However, few studies have fully used these identified factors to make RSB predictions.

Previous predictive studies on RSB have mostly been based on conventional regression models, which have high limitations of assumption and less ideal effects on RSB prediction. To fill such gaps, machine learning (ML) offers a possible alternative for factor identification and outcome prediction. In the past few years, a large number of studies have emerged using ML to predict the occurrence of STIs, which have achieved ideal performance [24,25]. However, RSB, as the main transmission route for STIs, has gained little attention from ML for prediction.

The objective of this study was to develop an ML-based model to precisely predict RSB in college students. Through a cross-province survey in China, this study aimed to develop a series of ML models to predict different types of RSB among Chinese college students. By comparison, we adopted the optimal model to identify key risk factors to help recognize and predict college students’ engagement in RSB, thus facilitating more precise intervention and prevention.

## Methods

### Participants and Research Procedures

We conducted a large-scale and internet-based survey, the National College Student Survey on Sexual and Reproductive Health in 2020 (NCSS-SRH 2020), sponsored by the China Family Planning Association (CFPA). Through multistage sampling from November 2019 to February 2020, a total of 55,757 Chinese college students from 241 universities completed the questionnaire survey. Voluntary participants were recruited using snowball sampling, and informed consent was obtained from each participant before completing the survey.

Among all participants, 1177 (2.11%) were excluded for either failing the attention check questions, ignoring the informed consent, or being outside the age range for college students (15-24 years old according to a standard definition of late adolescence and young adulthood by WHO) [26,27]. Samples with duplicated answers and variables with missing values over 5% were deleted. For the remaining variables, missing values were imputed with the use of multiple imputation. Due to the constraint of sexual orientation and past sexual experience, 8794 (15.78%) self-identified heterosexual students with sexual intercourse experience were finally included in the analyses.

### RSB Outcomes

We classified RSB from 4 perspectives: (1) whether contraception was used, (2) whether the contraceptive method was effective and safe, (3) whether participants engaged in casual sex or sex with multiple partners, and (4) the integration of the former 3 perspectives.

Contraception use was evaluated through 2 dimensions. Regarding frequency, contraception use was measured by the question “Do you use contraception while having sex every time you have sexual intercourse?” Regarding practices, contraception use was measured by the question “Did you/your partner use contraception the last time you had sex?” For both questions, a “no” response was considered to indicate RSB. According to WHO guidance, 4 contraceptive types with over 10 specific methods were investigated as contraception use: hormonal contraceptive methods, intrauterine devices, emergency contraception, and condoms [4].

The effectiveness and safety of contraception was mainly determined by the method of contraception. If someone reported using “emergency contraception,” “external ejaculation,” or a “safe period” for contraception, they were considered a member of the RSB group. The effectiveness and safety of contraception were also evaluated through the frequency and practice dimensions, which were, respectively, measured by the question of whether such approaches were taken every time or the last time of sexual intercourse.

Casual or multiple sex was assessed with the following questions: “Have you ever had sex through ‘booty calls’, ‘one-night stands’, ‘buying sex’, or ‘sex with multiple partners’?” As before, a “yes” response was considered RSB.

Finally, integrated RSB dimension was the combination of contraception use, the safety of contraception, and casual sex or sex with multiple partners. As long as 1 of these 3 types of behaviors occurred, we considered the student to have met the criteria for integrated RSB.

### Predictors

We included 126 potential predictors, including baseline characteristics (sex, age, ethnicity, religion, income, parental information, etc), daily habits (exercise, appearance and popularity, mobile phone addiction, alcohol and tobacco consumption, etc), physical and mental health status, relationship status, sexual knowledge, sexual education, sexual attitude, previous sexual experience, experience of sexual harassment and assault, etc. Among them, the age of the participant, the age of the participant’s partner, the income and expenditure of the participant, and the frequency of sports were treated as continuous variables. Other variables, including the degree of agreement, the frequency of participation, and the order of evaluation, were treated as either binary variables or ordered categorical variables.

Continuous variables were standardized, and categorical variables were split into multidimensional Boolean values before applying ML models. For each type of RSB, we used ML models to select and identify key variables to predict RSB among college students.

### Statistical Analyses

For descriptive statistics of baseline characteristics, continuous data were presented as the mean (SD) or the median (IQR), and the Student *t*-test or the Kruskal-Wallis test was applied depending on the normality distribution. Categorical data were presented in the form of counts with percentages, and the chi-square test or the Fisher exact test was applied. *P*<.05 in a 2-tailed test was considered statistically significant in these tests. In addition, we developed multivariable mixed models with a logit link function to learn the specific linear relationship between RSB and the key variables screened by ML. The results were presented in the form of point estimates of coefficients and corresponding 95% CIs, and statistical significance was accepted when *P*<.05.

### Model Development and Validation

We used 8 ML approaches: multiple logistic regression (MLR), naive Bayes (BYS), linear discriminant analysis (LDA), random forest (RF), gradient boosting machine (GBM), extreme gradient boosting (XGBoost), deep learning (DL), and the ensemble model. The ensemble model used the average values of all other models’ predictive values to perform classification. The data set was split into a training set and a test set randomly in a ratio of 8:2, with 7035 (80%) samples in the training set and 1759 (20%) samples in the test set. Our models were built on the training set and then applied to the test set for RSB prediction. Model discrimination was assessed through the receiver operator characteristic (ROC) curve, and model performance was assessed through accuracy, precision, recall, *F*_1_-score, the area under the receiver operator characteristic curve (AUROC), and the root-mean-square error (RMSE) calculated on the test set, which were presented in the form of the mean (SD).

Through comparison, we chose the optimal model for RSB prediction and included the most predictable variables in the model. The appropriate number of predictable variables was determined by the turning points of the model performance indicators. If all indicators did not change significantly through statistical testing when a new variable was added, we considered the turning point to have been reached.

To obtain the optimal performance for each model, we adopted the minimum distance (MD) method to select cut-off points to discriminate predictive values into 0 or 1. The MD method regarded the point closest to (0,1) on the ROC curve as the optimal cut-off point. To ensure the reliability and minimize the sensitivity of the results, we used a 10-fold cross-validation method to select the tuning hyperparameters as well as cut-off points. In addition, we repeated this process 10 times to minimize the influence of accidental circumstances.

All models were generated using R version 4.0.3 (R Core Team and the R Foundation for Statistical Computing). We used the *glmnet* package for MLR, the *e1071* package for BYS, the *MASS* package for LDA, the *randomForest* package for the RF, the *gbm* package for GBM, the *xgboost* package for XGBoost, and the *h20* package for DL.

### Ethical Considerations

Ethical approval was obtained from the Institutional Review Board of Tsinghua University (IRB no. 20190083). All participants provided informed consent online, which was set before answering the questionnaire and emphasized the autonomy of participating and the ability to withdraw at any time. The privacy of personal information was protected throughout the study via anonymous data collection, and confidentiality was maintained by asking participants to provide honest answers. Eligible participation in this survey was voluntary and was not compensated.

## Results

### Baseline Characteristics

The baseline characteristics of the participants are shown in Table 1. Our sample covered all provincial-level administrative regions in China and achieved a relatively good balance in the sample division of eastern, central, western, and northeastern regions (n=4758, 54.11%, n=1484, 16.88%, n=2140, 24.33%, and n=412, 4.69%, respectively), as well as the sex ratio (males: n=3918, 44.55%; females: n=4876, 55.45%). A total of 8794 students were included, among which 3682 (41.87%) did not use contraception every time they engaged in sexual intercourse, 3602 (40.96%) did not use safe or effective contraceptive methods every time they engaged in sexual intercourse, 1157 (13.16%) had casual sex or sex with multiple partners, and 5328 (60.59%) had experience with at least 1 of those 3 former behaviors before. In addition, 307 (3.49%) participants did not use contraception and 2140 (24.33%) used ineffective or unsafe contraceptive methods during their last sexual intercourse, the details of which are shown in Multimedia Appendix 1. The distribution of basic characteristics differed greatly in terms of RSB. Between the 2 groups with and without integrated RSB, the region of residence, sex, age, ethnicity, religious beliefs, urbanization of hometown, left-behind experience, migration experience, and self-assessment of family finances were significantly different (*P*<.05) among college students.

**Table 1:** Baseline characteristics of participants (N=8794) grouped by different types of RSB^a^.

Characteristics		Overall	RSB type 1 (nonuse of contraception)^b^	RSB type 2 (ineffective or unsafe contraceptive method)^c^	RSB type 3 (casual sex or sex with multiple partners)^d^	RSB type 4 (integrated RSB)^e^
**Region of residence, n (%); *P*_1_<.001, *P*_2_<.001, *P*_3_=.007, *P*_4_<.001^f^**						
	Eastern	4758 (54.11)	1770 (48.07)	1779 (49.39)	676 (58.43)	2697 (50.62)
	Central	1484 (16.88)	660 (17.93)	627 (17.41)	163 (14.09)	910 (17.08)
	Western	2140 (24.33)	1094 (29.71)	1026 (28.48)	263 (22.73)	1476 (27.70)
	Northeastern	412 (4.69)	158 (4.29)	170 (4.72)	55 (4.75)	245 (4.60)
**Sex, n (%); *P*_1_<.001, *P*_2_=.15, *P*_3_<.001, *P*_4_<.001**						
	Male	3918 (44.55)	1826 (49.59)	1638 (45.47)	646 (55.83)	2573 (48.29)
	Female	4876 (55.45)	1856 (50.41)	1964 (54.53)	511 (44.17)	2755 (51.71)
**Self-assessed gender-role conformity^g^, n (%); *P*_1_=.29, *P*_2_=.70, *P*_3_<.001, *P*_4_=.52**						
	Low	277 (3.15)	104 (2.82)	109 (3.03)	60 (5.19)	177 (3.32)
	Middle	4158 (47.28)	1759 (47.77)	1720 (47.75)	500 (43.22)	2515 (47.20)
	High	4359 (49.57)	1819 (49.40)	1773 (49.22)	597 (51.60)	2636 (49.47)
Age (years), median (IQR); *P*_1_<.001, *P*_2_<.001, *P*_3_<.001, *P*_4_<.001		20.00 (19.00-21.00)	20.00 (19.00-21.00)	20.00 (19.00-21.00)	20.00 (19.00-22.00)	20.00 (19.00-21.00)
**Ethnicity, n (%); *P*_1_<.001, *P*_2_<.001, *P*_3_=.77, *P*_4_<.001**						
	Han	7902 (89.86)	3240 (88.00)	3164 (87.84)	1043 (90.15)	4698 (88.18)
	Minority	892 (10.14)	442 (12.00)	438 (12.16)	114 (9.85)	630 (11.82)
**Religious beliefs, n (%); *P*_1_=.005, *P*_2_=.051, *P*_3_=.17, *P*_4_=.006**						
	No	8039 (91.41)	3329 (90.41)	3267 (90.70)	1045 (90.32)	4835 (90.75)
	Yes	755 (8.59)	353 (9.59)	335 (9.30)	112 (9.68)	493 (9.25)
Average monthly expenditure (CNY)/US $^h^, median (IQR); *P*_1_=.29, *P*_2_=.64, *P*_3_<.001, *P*_4_=.47		1800.00 (1200.00-2500.00)/265.48 (176.99-368.73)	1800.00 (1200.00-2500.00)/265.48 (176.99-368.73)	1800.00 (1200.00-2500.00)/265.48 (176.99-368.73)	2000.00 (1500.00-3000.00)/294.98 (221.24-442.47)	1800.00 (1200.00-2500.00)/265.48 (176.99-368.73)
**Urbanization of hometown, n (%); *P*_1_<.001, *P*_2_=.16, *P*_3_<.001, *P*_4_=.001**						
	Urban	4827 (54.89)	1893 (51.41)	1940 (53.86)	745 (64.39)	2846 (53.42)
	Suburban	2669 (30.35)	1166 (31.67)	1103 (30.62)	281 (24.29)	1644 (30.86)
	Rural	1298 (14.76)	623 (16.92)	559 (15.52)	131 (11.32)	838 (15.73)
**Left-behind experience, n (%); *P*_1_<.001, *P*_2_<.001, *P*_3_=.006, *P*_4_<.001**						
	No	6184 (70.32)	2426 (65.89)	2450 (68.02)	854 (73.81)	3631 (68.15)
	Yes	2610 (29.68)	1256 (34.11)	1152 (31.98)	303 (26.19)	1697 (31.85)
**Migration experience, n (%); *P*_1_=.04, *P*_2_<.001, *P*_3_=.14, *P*_4_=.003**						
	No	6864 (78.05)	2830 (76.86)	2738 (76.01)	923 (79.78)	4101 (76.97)
	Yes	1930 (21.95)	852 (23.14)	864 (23.99)	234 (20.22)	1227 (23.03)
**Self-assessment of family finances^i^, n (%); *P*_1_<.001, *P*_2_=.08, *P*_3_=.14, *P*_4_>=.005**						
	Low	652 (7.41)	316 (8.58)	293 (8.13)	89 (7.69)	434 (8.15)
	Middle	7167 (81.50)	2985 (81.07)	2904 (80.62)	921 (79.60)	4303 (80.76)
	High	975 (11.09)	381 (10.35)	405 (11.24)	147 (12.71)	591 (11.09)

^a^RSB: risky sexual behavior.

^b^Nonuse of contraception indicated that someone did not use contraception while having sex every time.

^c^Ineffective or unsafe contraception indicated that someone often used unsafe contraceptive methods (eg, emergency contraception, external ejaculation, and safe period).

^d^Casual sex or sex with multiple partners indicated that someone had engaged in casual sex or sex with multiple partners before.

^e^Integrated RSB was the combination of the former 3 types of RSB.

^f^*P*_1_, *P* value of RSB type 1; *P*_2_, *P* value of RSB type 2; *P*_3_, *P* value of RSB type 3; *P*_4_, *P* value of RSB type 4.

^g^Self-assessed gender role conformity is a 1-7–ordered categorical-scale question. We classified the responses into 3 groups: low conformity (1-2), middle conformity (3-5), and high conformity (6-7).

^h^CNY 1=US $0.145749.

^i^Self-assessment of family finances is a 1-7–ordered categorical-scale question. We classified the responses into 3 groups: low income (1-2), middle income (3-5), and high income (6-7).

### Model Performance and Validation

To precisely identify RSB among Chinese college students, we used various ML models to execute RSB prediction on the test data set to choose the optimal model. The model performance for the 4 types of RSB is presented in Table 2, and the model performance for the other 2 types of RSB based on the last sexual intercourse is presented in Multimedia Appendix 1. Through the comparison from multiple rounds of experiments, it was obvious that some ML models had better efficiency than the traditional multilinear logistic regression model. Compared to the performance of MLR (for which the average AUROCs were 0.76, 0.71, 0.91, and 0.79, respectively), XGBoost, GBM, and the RF presented better performance in terms of accuracy, the *F*_1_-score, and the AUROC.

**Figure 1:**
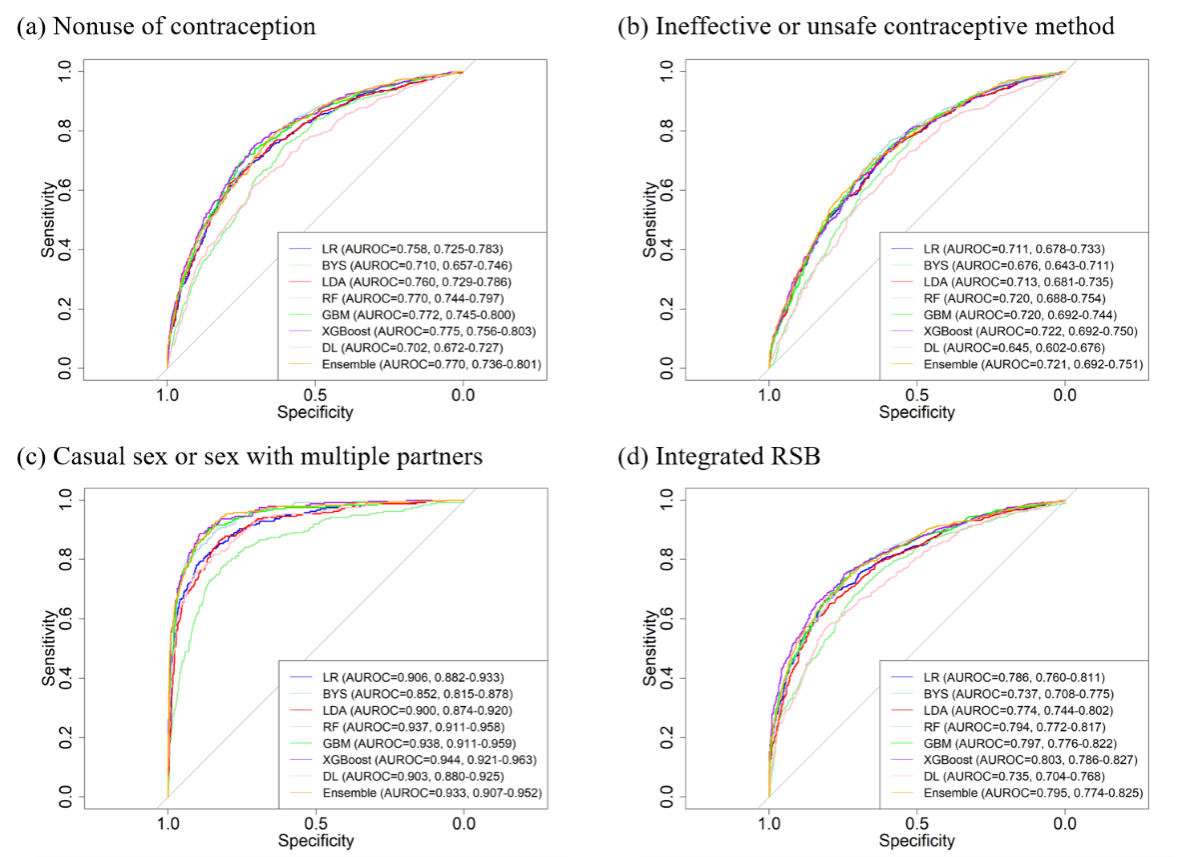
AUROC curves among the different types of RSB. AUROC: area under the receiver operator characteristic curve; BYS: naive Bayes; DL: deep learning; GBM: gradient boosting machine; LDA: linear discriminant analysis; LR: logistic regression; RF: random forest; RSB: risky sexual behavior; XGBoost: extreme gradient boosting.

To better understand model discrimination, we plotted ROC curves of all models on 4 types of RSB in Figure 1. Similarly, ROC curves of the other 2 types of RSB based on the last time of sexual intercourse are presented in Multimedia Appendix 1. It could be inferred that the curves of XGBoost and GBM were above the other curves, which suggests that these 2 models outperformed the others. Their average results were similar (average AUROCs were 0.77, 0.72, 0.94, and 0.80, respectively, in GBM and 0.78, 0.72, 0.94, and 0.80, respectively, in XGBoost). The ensemble model also played an effective role in predicting different types of RSB (average AUROCs were 0.77, 0.72, 0.93, and 0.80, respectively).

**Table 2:** Model performance among different types of RSB^a^.

RSB	Model	Accuracy, mean (SD)	Precision, mean (SD)	Recall, mean (SD)	F_1_-score, mean (SD)	AUROC^b^, mean (SD)	RMSE^c^, mean (SD)
**Nonuse of contraception^d^**							
	MLR^e^	0.70 (0.01)	0.63 (0.02)	0.70 (0.02)	0.66 (0.01)	0.76 (0.01)	0.44 (0.01)
	BYS^f^	0.66 (0.01)	0.58 (0.02)	0.68 (0.02)	0.62 (0.01)	0.71 (0.01)	0.53 (0.01)
	LDA^g^	0.70 (0.01)	0.63 (0.02)	0.71 (0.02)	0.66 (0.01)	0.76 (0.01)	0.44 (0.01)
	RF^h^	0.71 (0.01)	0.63 (0.02)	0.72 (0.02)	0.67 (0.01)	0.77 (0.01)	0.44 (0.00)
	GBM^i^	0.71 (0.01)	0.64 (0.02)	0.72 (0.02)	0.67 (0.01)	0.77 (0.01)	1.07 (0.01)
	XGBoost^j^	0.71 (0.01)	0.63 (0.02)	0.72 (0.03)	0.67 (0.01)	0.78 (0.01)	0.44 (0.01)
	DL^k^	0.65 (0.01)	0.58 (0.02)	0.65 (0.03)	0.61 (0.01)	0.70 (0.01)	0.52 (0.01)
	Ensemble	0.71 (0.01)	0.63 (0.02)	0.73 (0.02)	0.67 (0.01)	0.77 (0.01)	0.44 (0.01)
**Ineffective or unsafe contraceptive method^l^**							
	MLR	0.66 (0.01)	0.57 (0.02)	0.67 (0.02)	0.62 (0.01)	0.71 (0.01)	0.46 (0.01)
	BYS	0.63 (0.01)	0.54 (0.02)	0.65 (0.04)	0.59 (0.02)	0.68 (0.01)	0.53 (0.01)
	LDA	0.66 (0.01)	0.58 (0.02)	0.66 (0.03)	0.62 (0.01)	0.71 (0.01)	0.46 (0.01)
	RF	0.67 (0.01)	0.58 (0.02)	0.67 (0.03)	0.62 (0.01)	0.72 (0.01)	0.46 (0.00)
	GBM	0.67 (0.01)	0.59 (0.02)	0.66 (0.03)	0.62 (0.01)	0.72 (0.01)	1.08 (0.01)
	XGBoost	0.67 (0.01)	0.59 (0.02)	0.67 (0.03)	0.62 (0.01)	0.72 (0.01)	0.46 (0.00)
	DL	0.61 (0.01)	0.52 (0.02)	0.6 (0.04)	0.56 (0.02)	0.65 (0.02)	0.54 (0.01)
	Ensemble	0.67 (0.01)	0.58 (0.02)	0.67 (0.02)	0.62 (0.01)	0.72 (0.01)	0.46 (0.01)
**Casual sex or sex with multiple partners^m^**							
	MLR	0.83 (0.02)	0.44 (0.03)	0.84 (0.02)	0.57 (0.03)	0.91 (0.01)	0.26 (0.01)
	BYS	0.79 (0.02)	0.37 (0.03)	0.77 (0.03)	0.5 (0.02)	0.85 (0.01)	0.36 (0.01)
	LDA	0.83 (0.01)	0.43 (0.03)	0.85 (0.02)	0.57 (0.02)	0.90 (0.01)	0.27 (0.01)
	RF	0.87 (0.01)	0.5 (0.03)	0.87 (0.02)	0.64 (0.03)	0.94 (0.01)	0.24 (0.01)
	GBM	0.87 (0.01)	0.52 (0.03)	0.88 (0.02)	0.65 (0.02)	0.94 (0.01)	1.03 (0.00)
	XGBoost	0.88 (0.01)	0.53 (0.03)	0.88 (0.02)	0.66 (0.02)	0.94 (0.01)	0.23 (0.01)
	DL	0.83 (0.02)	0.43 (0.03)	0.83 (0.02)	0.57 (0.03)	0.90 (0.01)	0.29 (0.01)
	Ensemble	0.86 (0.02)	0.48 (0.03)	0.86 (0.02)	0.62 (0.03)	0.93 (0.01)	0.24 (0.01)
**Integrated RSB^n^**							
	MLR	0.72 (0.01)	0.80 (0.01)	0.71 (0.02)	0.75 (0.01)	0.79 (0.01)	0.43 (0.01)
	BYS	0.68 (0.01)	0.76 (0.01)	0.68 (0.03)	0.72 (0.02)	0.74 (0.01)	0.56 (0.01)
	LDA	0.71 (0.01)	0.79 (0.01)	0.70 (0.02)	0.74 (0.01)	0.77 (0.01)	0.44 (0.01)
	RF	0.73 (0.01)	0.80 (0.01)	0.73 (0.02)	0.77 (0.01)	0.79 (0.01)	0.43 (0.00)
	GBM	0.73 (0.01)	0.81 (0.01)	0.73 (0.02)	0.77 (0.01)	0.80 (0.01)	1.05 (0.01)
	XGBoost	0.73 (0.01)	0.81 (0.01)	0.73 (0.02)	0.77 (0.01)	0.80 (0.01)	0.43 (0.01)
	DL	0.67 (0.01)	0.77 (0.01)	0.66 (0.02)	0.71 (0.01)	0.73 (0.01)	0.5 (0.01)
	Ensemble	0.73 (0.01)	0.80 (0.01)	0.74 (0.02)	0.77 (0.01)	0.79 (0.01)	0.43 (0.01)

^a^RSB: risky sexual behavior.

^b^AUROC: area under the receiver operator characteristic curve.

^c^RMSE: root-mean-square error.

^d^Nonuse of contraception indicated that someone did not use contraception while having sex every time.

^e^MLR: multiple logistic regression.

^f^BYS: naive Bayes.

^g^LDA: linear discriminant analysis.

^h^RF: random forest.

^i^GBM: gradient boosting machine.

^j^XGBoost: extreme gradient boosting.

^k^DL: deep learning.

^l^Ineffective or unsafe contraception indicated that someone often used unsafe contraceptive methods (eg, emergency contraception, external ejaculation, and safe period).

^m^Casual sex or sex with multiple partners indicated that someone had engaged in casual sex or sex with multiple partners before.

^n^Integrated RSB was the combination of the former 3 types of RSB.

### Variable Selection and Prediction for RSB

**Figure 2:**
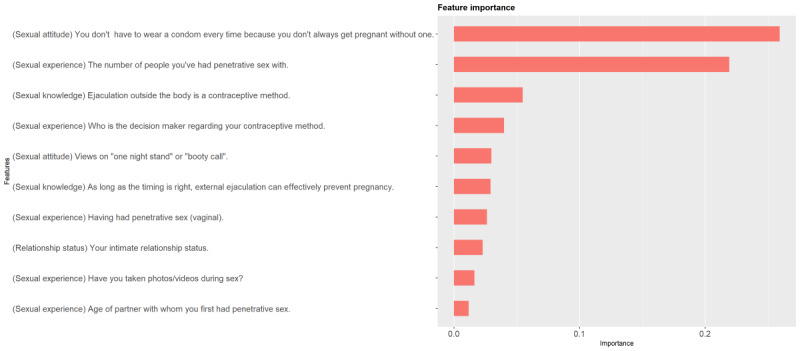
The 12 most predictive variables selected by the XGBoost model. XGBoost: extreme gradient boosting.

Through the comprehensive comparison of the evaluation indicators considering both efficiency and robustness, XGBoost was chosen to form the predicting model. To comprehensively predict RSB, we took integrated RSB as the outcome to select important variables, which were ranked in the order of importance according to the XGBoost model. Multimedia Appendix 1 shows the trend of 6 indicators as the number of variables increases, where points represent the average performance and lines represent the range. The turning points of the 6 indicators were 5th, 8th, 7th, 7th, 6th, and 12th, respectively. Thus, we finally chose 12 key variables for our prediction model, as presented in Figure 2. It could be inferred that RSB has multiple types of influencing factors, including relationship status, sexual knowledge, sexual attitudes, and previous sexual experience. The final prediction model we developed could estimate not only the probability of a student engaging in RSB but also the kind of RSB they were more likely to engage in.

To explore the specific association between the outcome variables and the predictive factors identified through XGBoost, we finally performed MLR, and the results of integrated RSB are presented in Table 3. It could be inferred that the lack of sexual knowledge and a liberal sexual attitude significantly increased the risk of RSB. In addition, regarding intimate relationship status, compared to the non-single group, the single group had a higher risk of RSB. Previous sexual experience also had a great influence on RSB. The greater the number of people participants had sexual intercourse with, the higher their risk of RSB. The more convenient the availability of contraceptives was, the less risk students would show RSB. The results of the other three types of RSB are shown in Multimedia Appendix 1.

**Table 3:** Association between integrated RSB^a^ and its key variables through MLR^b^.

Variable and option		Coefficient estimate		SE		*Z* value		*P* value (>|*Z*|)
**Sexual knowledge: ejaculation outside the body is a contraceptive method.**								
	Wrong	N/A^c^	N/A		N/A		N/A	
	Right	0.46	0.07		6.99		<.001	
**Sexual knowledge: having sex during a safe period is a contraceptive method.**								
	Wrong	N/A	N/A		N/A		N/A	
	Right	0.23	0.06		3.55		<.001	
**Sexual knowledge: as long as the timing is right, external ejaculation can effectively prevent pregnancy.**								
	Wrong	N/A	N/A		N/A		N/A	
	Right	0.42	0.06		–6.69		<.001	
**Sexual attitude: views on one-night stands or “booty calls.”**								
	I can accept it.	N/A	N/A		N/A		N/A	
	I can understand my friends doing this, but I cannot.	–0.44	0.07		–6.38		<.001	
	Totally unacceptable.	–0.28	0.07		–3.73		<.001	
**Sexual attitude: you don't have to wear a condom every time, because you don't always get pregnant without one.**								
	Strongly disagree	N/A	N/A		N/A		N/A	
	Relatively disagree	1.05	0.07		15.09		<.001	
	Not sure	1.28	0.11		11.66		<.001	
	Relatively agree	1.68	0.17		9.76		<.001	
	Strongly agree	1.25	0.25		5.07		<.001	
**Relationship status: your intimate relationship status.**								
	Single	N/A	N/A		N/A		N/A	
	Nonsingle	–0.39	0.06		–6.63		<.001	
**Sexual experience: having had penetrative sex (vaginal).**								
	Never has been and never will be acceptable.	N/A	N/A		N/A		N/A	
	Never has been but I can accept it in the future.	0.97	1.49		0.65		.52	
	I started before junior high school.	1.42	1.42		1.00		.32	
	I started since senior high school.	1.15	1.41		0.82		.42	
	I started since college.	0.81	1.41		0.57		.57	
**Sexual experience: have you taken photos/videos during sex?**								
	Never has been and never will be acceptable.	N/A	N/A		N/A		N/A	
	Never has been but I can accept it in the future.	0.01	0.07		0.16		.87	
	I started before junior high school.	0.17	0.35		0.49		.62	
	I started since senior high school.	0.45	0.13		3.48		.001	
	I started since college.	0.46	0.07		6.32		<.001	
Sexual experience: age of partner with whom you first had penetrative sex.		0.01		0.01		1.38		.17
Sexual experience: the number of people you've had penetrative sex with.		0.33		0.02		13.93		<.001
**Sexual experience: who is the decision maker regarding your contraceptive method?**								
	Myself.	N/A	N/A		N/A		N/A	
	My partner.	0.17	0.09		2.01		.04	
	By mutual negotiation.	0.11	0.06		1.82		.07	
	It depends.	1.30	0.13		10.06		<.001	
	Just use what we can find.	0.84	0.23		3.67		<.001	
	Others.	–0.31	0.33		–0.93		.36	
**Sexual experience: availability of contraceptives.**								
	Very convenient	N/A	N/A		N/A		N/A	
	Relatively convenient	0.30	0.06		5.41		<.001	
	Relatively inconvenient	0.41	0.08		4.91		<.001	
	Very inconvenient	0.28	0.14		2.02		.04	

^a^RSB: risky sexual behavior.

^b^MLR: multiple logistic regression.

^c^N/A: not applicable as the baseline group.

## Discussion

### Principal Results

This study validated the effectiveness of ML models in predicting RSB among college students through comparisons of multiple models. Among various ML models, XGBoost performed the best in this task, with a higher accuracy, precision, *F*_1_-score, and AUROC performance than others. Thus, we eventually used XGBoost to identify the 12 most predictive factors for total RSB, including relationship status, sexual knowledge, sexual attitudes, and previous sexual experience. This systematic process of data modeling as well as the accuracy of the final results indicated that ML approaches could have considerable value in RSB prediction and intervention among college students.

ML models were substantially superior to conventional regressions and should be recommended for more practical applications. Compared to the AUROC values of the MLR model (0.76, 0.71, 0.91, and 0.79 on the 4 types of RSB, respectively), XGBoost had a much higher effect in terms of AUROC values (0.78, 0.72, 0.94, and 0.80 on the 4 types of RSB, respectively). However, the capacity of explanation of MLR was nonnegligible. Thus, we finally used an MLR model again to investigate the linear association between outcomes and those important risk factors identified through XGBoost.

In this study, demographic characteristics and socioeconomic status were found to be significant factors of RSBs. Adolescents who were from an ethnic minority background, held religious beliefs, or had a lower family financial status tended to engage in more RSBs. This may be due to the lack of sexual education resources or specific religious customs. In addition, it was worth noting that students who had migration experience or were from rural hometowns were associated with increased RSBs. This finding was consistent with previous research and reflects the persisting gap in adolescent sexual and reproductive health between urban and rural areas [28,29]. Correlated with a lower level of education and socioeconomic status, rural-to-urban adolescents had less exposure to sexual knowledge and sex education [28,30].

In accordance with previous studies, romantic relationships were highly associated with RSB. It was found that college students with a romantic relationship had a significantly higher probability of using condoms during vaginal sex, oral sex, and anal sex [23]. On the one hand, students not in romantic relationships usually had fewer condom-carrying practices and a higher occurrence of unplanned sex. On the other hand, according to Rosenthal et al [31], rather than being concerned with the risk of STIs, students not in romantic relationships paid more attention to building intimacy through RSB, especially during casual sexual encounters. This is a worrisome mechanism since these students are more inclined to be unaware of each other’s health status, which could lead to a considerable risk of STIs.

This study also validated the role of sexual attitude in RSB. The more tolerant the students were toward condom nonuse and one-night stand, the higher their probability of engaging in RSB. In fact, it was widely validated that sexual attitude plays the most predictive influence in predicting RSB [32,33], which could be illustrated through the theory of planned behavior (TPB) that attitude is an activator of behavior [34]. Thus, sexual education should be facilitated to emphasize the importance and necessity of safe sex and dispel misunderstandings about it.

In addition, students’ previous sexual experience also had a large influence on the possibility of RSB, such as the number of sexual partners, the decision maker regarding the contraceptive method, and whether to take photos/videos during sex. This finding is also rational since well-practiced behavior would more likely recur due to the natural automation of initiates and controls [35].

### Limitations

There are some limitations in this study. First, all outcomes and predictors were self-reported by participants, which may have caused recall bias and nonresponse bias. Since some questions were sensitive, participants may not have been willing to provide correct answers, such as on previous sexual experience, experience of sexual harassment, and assault. Second, the measurement of contraception use did not differentiate between the types of contraceptive methods used by the study population. Different contraceptive methods serve different functions. Condom use can prevent both STIs and unwanted pregnancies, while hormonal contraception is only effective for pregnancy prevention. Although the vast majority of participants used condoms for contraception, the predictive accuracy of contraceptive effectiveness may still have been obscured in this study because of the lack of differentiation across methods. Third, the definition of casual sex and sex with multiple partners was not well specified. Though these 2 types of behavior were undoubtedly validated as RSBs, the risks of contracting STIs and unwanted pregnancy can be kept relatively low with correct condom use. Fourth, although ML models have better predictive performance than traditional regression models, their explanatory performance is much weaker. We performed MLR to compensate for this drawback, but multicollinearity may exist among those selected risk factors, which could lead to inaccurate estimation. In particular, we had a large scale of many questions, with much similarity among them. Thus, questions could be divided into clusters to decrease the variable dimensions as well as strengthen the explanatory power. Fifth, since our model relied on a cross-sectional questionnaire, the outcomes and predictors were questioned concurrently. Under the same time window, it is difficult to identify the sequence of events. There may be causal inversion problems between predictors and outcomes, and thus, the prospective predictive efficiency of the model is hard to validate.

### Comparison With Prior Work

There are considerable methodological, theoretical, and practical implications of this study. From the methodological aspect, we adopted a scientific and rigorous process to generate an RSB-predictive model using ML methods, which constitutes a research gap and urgent work to be done. The selection of the model, the adjustment of parameters, the comparison of indicators, and the finalization of variable numbers are of high reference value in the methodology. From a theoretical perspective, we identified a series of risk factors for RSB. We provided additional evidence for the association of demographic characteristics and socioeconomic status with RSB. Critical factors influencing RSB were also explored, including sexual attitude, sexual knowledge, relationship status, and sexual experience. Through the results presented, a comprehensive and evidence-based guideline was formed to facilitate more precise interventions and prevent RSB among adolescents and young adults. From a practical perspective, we developed a predictive model to help identify RSB among college students. Due to privacy concerns and the stigmatization of sexual behavior, it is often difficult to investigate the real prevalence of RSB among adolescents. Using the 12 predictors identified here, the model can predict not only the probability of a student engaging in RSB but also what kind of RSB they are more likely to engage in. With such a model, our study allows for more targeted intervention and prevention of RSB in students before they contract STIs, and thus, these students will be better able to avoid the various negative consequences of RSB, including STIs and unwanted pregnancy.

### Conclusion

In summary, our study confirmed that ML approaches, especially XGBoost, have greater predictive effects for RSB than traditional regression models. Such ML-based assessment tools could generate new applications with considerable practical value, which would promote health at both the individual and the public level in the future.

## Appendix

Multimedia Appendix 1 Supplementary materials.

## Abbreviations

AUROC: area under the receiver operator characteristic curve
BYS: naive Bayes
DL: deep learning
GBM: gradient boosting machine
LDA: linear discriminant analysis
MD: minimum distance
ML: machine learning
MLR: multiple logistic regression
RF: random forest
ROC: receiver operator characteristic
RMSE: root-mean-square error
RSB: risky sexual behavior
STI: sexually transmitted infection
WHO: World Health Organization
XGBoost: extreme gradient boosting

## Data Availability

The data that support the findings of this study are available upon reasonable request from the corresponding author (KT). The data are not publicly available due to research ethics board restrictions.

## References

[1] Keto T, Tilahun A, Mamo A (2020). Knowledge, attitude and practice towards risky sexual behaviors among secondary and preparatory students of Metu town, south western Ethiopia. BMC Public Health.

[2] Bellizzi S, Pichierri G, Menchini Le, Barry J, Sotgiu G, Bassat Q (2019). The impact of underuse of modern methods of contraception among adolescents with unintended pregnancies in 12 low- and middle-income countries. J Glob Health.

[3] World Health Organization Adolescent Pregnancy.

[4] World Health Organization Contraception.

[5] Apter D (2018). Contraception options: aspects unique to adolescent and young adult. Best Pract Res Clin Obstet Gynaecol.

[6] Pan X, Cong L, Ma Q, Xu G, Yu F, Zou Y (2006). [Perception on AIDS infection risk and condom use among 2785 college students having had sexual experience in Zhejiang Province]. Zhonghua Liu Xing Bing Xue Za Zhi.

[7] Florence C (2012). Reported Condom Use in Students Enrolled in a Personal Health and Wellness Course.

[8] Garga S, Thomas M, Bhatia A, Sullivan A, John-Leader F, Pit S (2021). Geosocial networking dating app usage and risky sexual behavior in young adults attending a music festival: cross-sectional questionnaire study. J Med Internet Res.

[9] Thornton LC, Frick PJ, Ray JV, Wall Myers TD, Steinberg L, Cauffman E (2019). Risky sex, drugs, sensation seeking, and callous unemotional traits in justice-involved male adolescents. J Clin Child Adolesc Psychol.

[10] Homma Y, Wang N, Saewyc E, Kishor N (2012). The relationship between sexual abuse and risky sexual behavior among adolescent boys: a meta-analysis. J Adolesc Health.

[11] Longo LM, Ertl MM, Pazienza R, Agiliga AU, Dillon FR, Martin JL (2020). Associations among negative urgency, sensation seeking, alcohol use, self-esteem, and casual sexual behavior for college students. Subst Use Misuse.

[12] Owen J, Fincham FD, Moore J (2011). Short-term prospective study of hooking up among college students. Arch Sex Behav.

[13] Anteneh ZA (2013). Prevalence and correlates of multiple sexual partnerships among private college students in Bahir Dar City, Northwest Ethiopia. Sci J Public Health.

[14] Medina-Perucha L, Family H, Scott J, Chapman S, Dack C (2019). Factors associated with sexual risks and risk of STIs, HIV and other blood-borne viruses among women using heroin and other drugs: a systematic literature review. AIDS Behav.

[15] Menon J, Mwaba S, Thankian K, Lwatula C (2016). Risky sexual behaviour among university students. Int STD Res Rev.

[16] Somba MJ, Mbonile M, Obure J, Mahande MJ (2014). Sexual behaviour, contraceptive knowledge and use among female undergraduates' students of Muhimbili and Dar es Salaam Universities, Tanzania: a cross-sectional study. BMC Womens Health.

[17] Yi S, Tuot S, Yung K, Kim S, Chhea C, Saphonn V (2014). Factors associated with risky sexual behavior among unmarried most-at-risk young people in Cambodia. AJPHR.

[18] Adeoti YF (2016). Predisposing Factors Influencing Risky Sexual Behaviours as Expressed by Undergraduates in Osun State Nigeria.

[19] Amare T, Yeneabat T, Amare Y (2019). A systematic review and meta-analysis of epidemiology of risky sexual behaviors in college and university students in Ethiopia. J Environ Public Health.

[20] Jackson JM, Seth P, DiClemente RJ, Lin A (2015). Association of depressive symptoms and substance use with risky sexual behavior and sexually transmitted infections among African American female adolescents seeking sexual health care. Am J Public Health.

[21] Vasilenko SA, Kugler KC, Butera NM, Lanza ST (2015). Patterns of adolescent sexual behavior predicting young adult sexually transmitted infections: a latent class analysis approach. Arch Sex Behav.

[22] Long Lu, Han Y, Tong L, Chen Z (2019). Association between condom use and perspectives on contraceptive responsibility in different sexual relationships among sexually active college students in China: a cross-sectional study. Medicine (Baltimore).

[23] Ssewanyana D, Sebena R, Petkeviciene J, Lukács A, Miovsky M, Stock C (2015). Condom use in the context of romantic relationships: a study among university students from 12 universities in four Central and Eastern European countries. Eur J Contracept Reprod Health Care.

[24] Bao Y, Medland NA, Fairley CK, Wu J, Shang X, Chow EP, Xu X, Ge Z, Zhuang X, Zhang L (2021). Predicting the diagnosis of HIV and sexually transmitted infections among men who have sex with men using machine learning approaches. J Infect.

[25] Feller D, Zucker J, Yin MT, Gordon P, Elhadad N (2018). Using clinical notes and natural language processing for automated HIV risk assessment. J Acquir Immune Defic Syndr.

[26] Gore FM, Bloem PJ, Patton GC, Ferguson J, Joseph V, Coffey C, Sawyer SM, Mathers CD (2011). Global burden of disease in young people aged 10–24 years: a systematic analysis. Lancet.

[27] World Health Organization Older Adolescent (15 to 19 years) and Young Adult (20 to 24 years) Mortality.

[28] Sudhinaraset M, Astone N, Blum RW (2012). Migration and unprotected sex in Shanghai, China: correlates of condom use and contraceptive consistency across migrant and nonmigrant youth. J Adolesc Health.

[29] Li S, Huang H, Cai Y, Xu G, Huang F, Shen X (2009). Characteristics and determinants of sexual behavior among adolescents of migrant workers in Shangai (China). BMC Public Health.

[30] Zhao S, Gao E, Zabin LS (2008). Unmet needs for reproductive health knowledge among unmarried migrant youth. J Reprod Contracept.

[31] Rosenthal D, Gifford S, Moore S (1998). Safe sex or safe love: competing discourses?. AIDS Care.

[32] McEachan RRC, Conner M, Taylor NJ, Lawton RJ (2011). Prospective prediction of health-related behaviours with the theory of planned behaviour: a meta-analysis. Health Psychol Rev.

[33] Albarracín D, Johnson BT, Fishbein M, Muellerleile PA (2001). Theories of reasoned action and planned behavior as models of condom use: a meta-analysis. Psychol Bull.

[34] Bargh JA, Chen M, Burrows L (1996). Automaticity of social behavior: direct effects of trait construct and stereotype activation on action. J Pers Soc Psychol.

[35] Ouellette JA, Wood W (1998). Habit and intention in everyday life: the multiple processes by which past behavior predicts future behavior. Psychol Bull.

