# Supplementary material for: Predicting Risky Sexual Behavior Among College Students Through Machine Learning Approaches: Cross-sectional Analysis of Individual Data From 1264 Universities in 31 Provinces in China
Source: JMIR Public Health Surveill. 2023 Jan 25;9:e41162. doi: 10.2196/41162 (PMC9909517; doi:10.2196/41162)
Supplement: Multimedia Appendix 1 [file publichealth_v9i1e41162_app1.pdf]

Multimedia Appendix 1

Table S1 Baseline characteristics of participants grouped by different types of RSB during their last sexual intercourse

		Whether contraception wasn't used last time		Whether contraception wasn't safe last time	
		Yes (n=307)	P value	Yes (n=2140)	P value
Region of residence					
	Eastern	130 (42.35)	<.001	1011 (47.24)	<.001
	Central	69 (22.48)		403 (18.83)	
	Western	90 (29.32)		641 (29.95)	
	Northeastern	18 (5.86)		85 (3.97)	
Sex					
	Male	146 (47.56)	.006	1011 (47.24)	0·001
	Female	161 (52.44)		403 (18.83)	
Self-assessed gender-role conformity [a]					
	Low	9 (2.93)	.90	65 (3.04)	.71
	Middle	149 (48.53)		1028 (48.04)	
	High	149 (48.53)		1047 (48.93)	
Age					
		19.00 [19.00, 21.00]	<.001	20.00 [19.00, 21.00]	<.001
Ethnicity					
	Han	273 (88.93)	.65	1852 (86.54)	<.001
	Minority	34 (11.07)		288 (13.46)	
Religious beliefs					
	No	280 (91.21)	.98	1920 (89.72)	.002
	Yes	27 (8.79)		220 (10.28)	
Average monthly expenditure (Yuan)					
		1800.00 [1275.00, 2750.00]	.50	1500.00 [1200.00, 2000.00]	<.001
Urbanization of hometown					
	Urban	162 (52.77)	.22	1095 (51.17)	<.001
	Suburban	89 (28.99)		694 (32.43)	
	Rural	56 (18.24)		351 (16.40)	
Left-behind experience					
	No	198 (64.50)	.03	1433 (66.96)	<0·001
	Yes	109 (35.50)		707 (33.04)	
Migration experience					
	No	234 (76.22)	.47	1645 (76.87)	.14
	Yes	73 (23.78)		495 (23.13)	
Self-assessment of family finances [b]					
	Low	28 (9.12)	.25	171 (7.99)	.41
	Middle	252 (82.08)		1725 (80.61)	
	High	27 (8.79)		244 (11.40)	

Data are presented in the form of n (%) or median (IQR).

a. Self-assessed gender-role conformity is a 1-7 ordered categorical scale question. We classified the responses into three groups: low conformity (1-2), middle conformity (3-5) and high conformity (6-7).

b. Self-assessment of family finances is a 1-7 ordered categorical scale question. We classified the responses into three groups: low-income (1-2), middle income (3-5) and high income (6-7).

Table S2 Model performance among different types of RSB during their last sexual intercourse

RSB	Model	Accuracy	Precision	Recall	F1	AUROC[a]	RMSE[b]
Whether contraception wasn't used last time	MLR[c]	0.63 (0.06)	0.98 (0)	0.63 (0.07)	0.76 (0.05)	0.62 (0.03)	0.19 (0.01)
	BYS[d]	0.64 (0.05)	0.98 (0)	0.64 (0.06)	0.77 (0.04)	0.68 (0.03)	0.3 (0.01)
	LDA[e]	0.63 (0.05)	0.98 (0)	0.63 (0.06)	0.76 (0.04)	0.65 (0.03)	0.2 (0.01)
	RF[f]	0.65 (0.05)	0.98 (0)	0.64 (0.06)	0.78 (0.04)	0.7 (0.03)	0.24 (0.01)
	GBM[g]	0.66 (0.04)	0.98 (0)	0.66 (0.04)	0.79 (0.03)	0.69 (0.03)	1 (0.01)
	XGB[h]	0.66 (0.04)	0.98 (0)	0.66 (0.04)	0.79 (0.03)	0.7 (0.03)	0.2 (0.01)
	DLi	0.6 (0.07)	0.97 (0)	0.6 (0.07)	0.74 (0.05)	0.59 (0.03)	0.21 (0.01)
	Ensemble	0.64 (0.04)	0.98 (0)	0.63 (0.04)	0.77 (0.03)	0.71 (0.03)	0.2 (0.01)
Whether contraception was effective and safe last time	MLR[c]	0.66 (0.02)	0.38 (0.02)	0.66 (0.03)	0.49 (0.01)	0.71 (0.01)	0.41 (0.01)
	BYS[d]	0.62 (0.02)	0.35 (0.02)	0.65 (0.03)	0.45 (0.02)	0.67 (0.01)	0.52 (0.01)
	LDA[e]	0.66 (0.02)	0.38 (0.02)	0.67 (0.03)	0.49 (0.01)	0.71 (0.01)	0.41 (0.01)
	RF[g]	0.66 (0.02)	0.39 (0.02)	0.68 (0.03)	0.49 (0.01)	0.72 (0.01)	0.45 (0.01)
	GBM[h]	0.67 (0.02)	0.39 (0.02)	0.68 (0.03)	0.49 (0.01)	0.72 (0.01)	1.1 (0.01)
	XGB[i]	0.67 (0.01)	0.39 (0.02)	0.68 (0.03)	0.5 (0.01)	0.72 (0.01)	0.41 (0.01)
	DL[j]	0.61 (0.02)	0.33 (0.02)	0.6 (0.03)	0.43 (0.02)	0.64 (0.02)	0.48 (0.01)
	Ensemble	0.66 (0.02)	0.39 (0.02)	0.68 (0.04)	0.49 (0.02)	0.72 (0.01)	0.41 (0.01)

Data are presented as the mean (SD).

a. AUROC is area under the receiver operator characteristic curve.

b. RMSE is root mean square error.

c. MLR is multiple logistic regression.

d. BYS is naive bayes.

e. LDA is linear discriminant analysis.

f. RF is random forest.

g. GBM is gradient boosting machine.

h. XGB is XGBoost.

i. DL is deep learning.

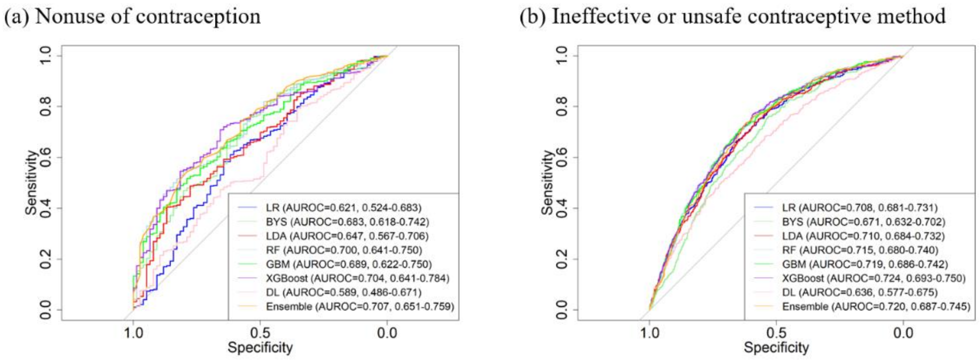

Figure S1 AUROC curves among the different types of RSB during their last sexual intercourse

a. Nonuse of contraception indicated that someone did not use contraception while having sex every time.

b. Unsafe contraception method indicated that someone often used unsafe contraceptive methods (including emergency contraception, external ejaculation, and safe period).

c. AUROC is the area under the receiver operator characteristic curve.

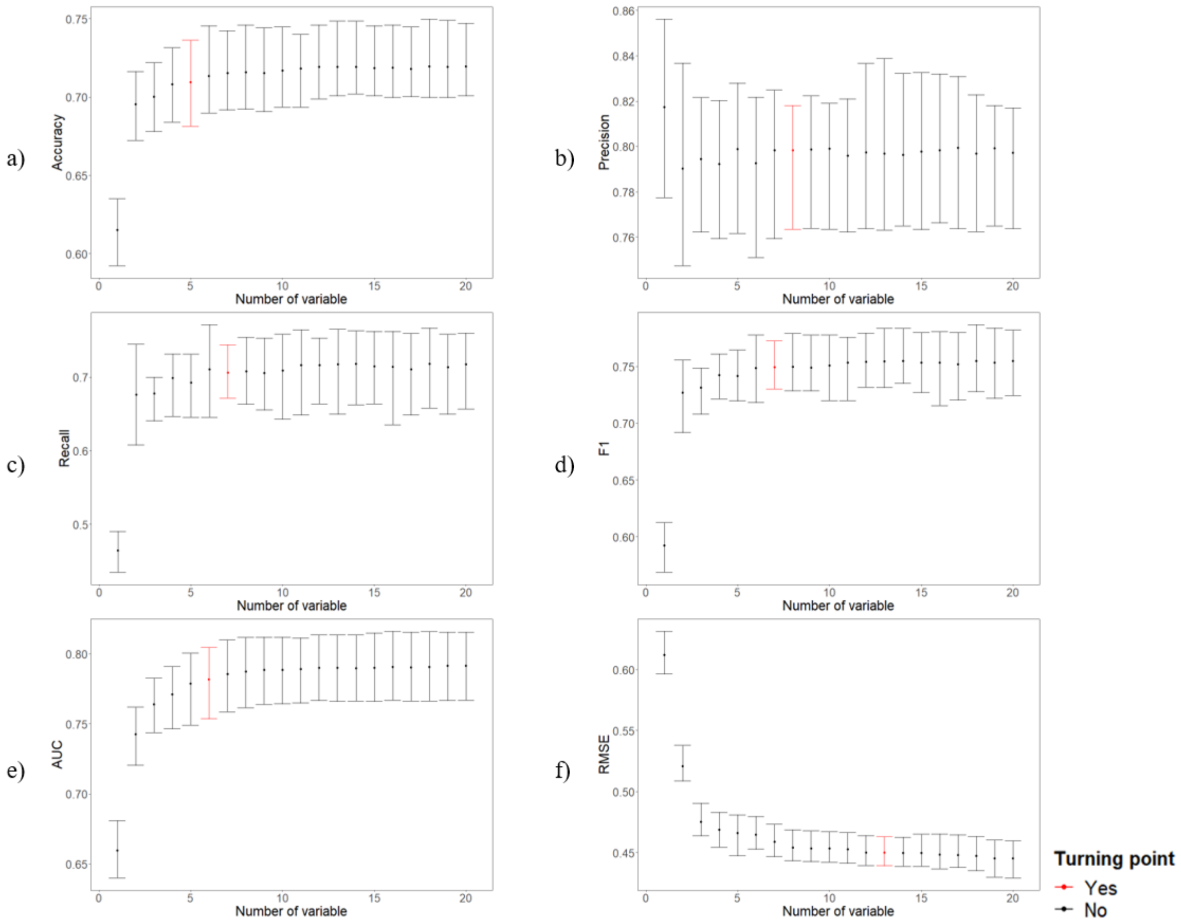

Figure S2 The trend of six indicators as the number of variables increases

Table S3 Association between nonuse of contraception and its key variables through logistic regression

Variable	Option	Coefficient estimate	Std. Error	z value	Pr(>|z|)
(Intercept)		-0.04	1.34	-0.03	.98
(Sexual knowledge) Ejaculation outside the body is a contraceptive method.					
	Wrong	-	-	-	-
	Right	0.28	0.06	4.49	<.001
(Sexual knowledge) Having sex during a safe period is a contraceptive method.					
	Wrong	-	-	-	-
	Right	0.13	0.06	2.02	.04
(Sexual knowledge) As long as the timing is right, external ejaculation can effectively prevent pregnancy.					
	Wrong	-	-	-	-
	Right	-0.12	0.06	-2.01	.045
(Sexual attitude) You don't have to wear a condom every time because you don't always get pregnant without one.					
	Strongly disagree	-	-	-	-
	Relatively disagree	1.15	0.06	18.89	<.001
	Not sure	1.48	0.09	16.48	<.001
	Relatively agree	1.81	0.13	14.41	<.001
	Strongly agree	1.35	0.18	7.42	<.001
(Relationship status) Your intimate relationship status.					
	Single	-	-	-	-
	Non-single	-0.19	0.05	-3.41	.001
(Sexual experience) Having had penetrative sex (vaginal).					
	Never has been and never will be acceptable.	-	-	-	-
	Never has been but I can accept it in the future.	-0.76	1.42	-0.53	.59
	I started before junior high school.	0.15	1.33	0.11	.91
	I started since senior high school.	-0.35	1.33	-0.26	.79
	I started since college.	-0.77	1.33	-0.58	.56
(Sexual experience) Have you taken photos/videos during sex?					
	Never has been and never will be acceptable.	-	-	-	-
	Never has been but I can accept it in the future.	-0.06	0.07	-0.80	.42
	I started before junior high school.	-0.47	0.26	-1.83	.07
	I started since senior high school.	0.41	0.11	3.83	<.001
	I started since college.	0.53	0.07	7.64	<.001
(Sexual experience) Age of partner with whom you first had penetrative sex.					
		-0.03	0.01	-3.15	.002
(Sexual experience) The number of people you've had penetrative sex with.					
		0.02	0.00	3.35	.001

(Sexual attitude) Views on "one night stand" or "booty call".				
I can accept it.	-	-	-	-
I can understand my friends doing this, but I can't.	-0.22	0.06	-3.55	<.001
Totally unacceptable.	-0.11	0.07	-1.60	.11
(Sexual experience) Who is the decision maker regarding your contraceptive method.				
Myself.	-	-	-	-
My partner.	0.13	0.08	1.51	.13
By mutual negotiation.	0.01	0.06	0.22	.82
It depends.	1.09	0.10	10.65	<.001
Just use what we can find.	0.34	0.18	1.84	.07
Others.	-0.33	0.34	-0.98	.33
(Sexual experience) Availability of contraceptives.				
Very convenient.	-	-	-	-
Relatively convenient.	0.32	0.05	5.91	<.001
Relatively inconvenient.	0.49	0.08	6.13	<.001
Very inconvenient.	0.45	0.13	3.38	.001

Table S4 Association between ineffective or unsafe contraceptive method and its key variables through logistic regression

Variable	Option	Coefficient estimate	Std. Error	z value	Pr(>|z|)
(Intercept)		-13.14	111.34	-0.12	.91
(Sexual knowledge) Ejaculation outside the body is a contraceptive method.					
	Wrong	-	-	-	-
	Right	0.56	0.06	9.31	<.001
(Sexual knowledge) Having sex during a safe period is a contraceptive method.					
	Wrong	-	-	-	-
	Right	0.42	0.06	7.19	<.001
(Sexual knowledge) As long as the timing is right, external ejaculation can effectively prevent pregnancy.					
	Wrong	-	-	-	-
	Right	0.32	0.06	-5.78	<.001
(Sexual attitude) You don't have to wear a condom every time because you don't always get pregnant without one.					
	Strongly disagree	-	-	-	-
	Relatively disagree	0.63	0.06	10.57	<.001
	Not sure	0.63	0.08	7.44	<.001
	Relatively agree	0.89	0.11	7.96	<.001
	Strongly agree	0.50	0.17	3.01	.003
(Relationship status) Your intimate relationship status.					
	Single	-	-	-	-
	Non-single	-0.11	0.05	-1.99	.047
(Sexual experience) Having had penetrative sex (vaginal).					
	Never has been and never will be acceptable.	-	-	-	-
	Never has been but I can accept it in the future.	10.65	111.34	0.10	.92
	I started before junior high school.	11.90	111.33	0.11	.91
	I started since senior high school.	11.83	111.33	0.11	.91
	I started since college.	11.55	111.33	0.10	.92
(Sexual experience) Have you taken photos/videos during sex?					
	Never has been and never will be acceptable.	-	-	-	-
	Never has been but I can accept it in the future.	-0.04	0.07	-0.54	.59
	I started before junior high school.	0.19	0.24	0.78	.44
	I started since senior high school.	0.56	0.10	5.45	<.001
	I started since college.	0.47	0.07	7.02	<.001
(Sexual experience) Age of partner with whom you first had penetrative sex.					
		0.01	0.01	1.00	.32
(Sexual experience) The number of people you've had penetrative sex with.					
		0.01	0.00	1.64	.10
(Sexual attitude) Views on "one night stand" or "booty call".					
	I can accept it.	-	-	-	-
	I can understand my friends doing this, but I can't.	-0.09	0.06	-1.53	.13
	Totally unacceptable.	-0.03	0.07	-0.44	.66
(Sexual experience) Who is the decision maker regarding your contraceptive method.					

Myself.	-	-	-	-
My partner.	0.22	0.08	2.76	.006
By mutual negotiation.	0.22	0.06	3.86	<.001
It depends.	0.88	0.10	9.26	<.001
Just use what we can find.	0.42	0.18	2.36	.02
Others.	-0.98	0.39	-2.54	.01
(Sexual experience) Availability of contraceptives.				
Very convenient.	-	-	-	-
Relatively convenient.	0.23	0.05	4.35	<.001
Relatively inconvenient.	0.32	0.08	4.17	<.001
Very inconvenient.	0.12	0.13	0.95	.34

Table S5 Association between Casual sex or sex with multiple partners and its key variables through logistic regression

Variable	Option	Coefficient estimate	Std. Error	z value	Pr(>|z|)
(Intercept)		-15.88	305.78	-0.05	.96
(Sexual knowledge) Ejaculation outside the body is a contraceptive method.					
	Wrong	-	-	-	-
	Right	0.13	0.11	1.18	.24
(Sexual knowledge) Having sex during a safe period is a contraceptive method.					
	Wrong	-	-	-	-
	Right	-0.27	0.11	-2.55	.01
(Sexual knowledge) As long as the timing is right, external ejaculation can effectively prevent pregnancy.					
	Wrong	-	-	-	-
	Right	0.23	0.10	2.27	.02
(Sexual attitude) You don't have to wear a condom every time because you don't always get pregnant without one.					
	Strongly disagree	-	-	-	-
	Relatively disagree	-0.05	0.10	-0.52	.60
	Not sure	-0.18	0.15	-1.21	.23
	Relatively agree	-0.07	0.18	-0.37	.71
	Strongly agree	-0.01	0.25	-0.02	.98
(Relationship status) Your intimate relationship status.					
	Single	-	-	-	-
	Non-single	-0.50	0.08	-6.03	<.001
(Sexual experience) Having had penetrative sex (vaginal).					
	Never has been and never will be acceptable.	-	-	-	-
	Never has been but I can accept it in the future.	11.85	305.78	0.04	.97
	I started before junior high school.	13.09	305.78	0.04	.97
	I started since senior high school.	12.87	305.78	0.04	.97
	I started since college.	12.65	305.78	0.04	.97
(Sexual experience) Have you taken photos/videos during sex?					
	Never has been and never will be acceptable.	-	-	-	-
	Never has been but I can accept it in the future.	0.30	0.11	2.78	.005
	I started before junior high school.	1.19	0.33	3.59	<.001
	I started since senior high school.	0.56	0.15	3.70	<.001
	I started since college.	0.76	0.11	6.98	<.001
(Sexual experience) Age of partner with whom you first had penetrative sex.					
		0.07	0.01	4.87	<.001
(Sexual experience) The number of people you've had penetrative sex with.					
		0.30	0.02	17.88	<.001
(Sexual attitude) Views on 'one night stand' or 'booty call'.					
	I can accept it.	-	-	-	-

	I can understand my friends doing this, but I can't.	-2.25	0.11	-20.50	<.001
	Totally unacceptable.	-2.52	0.15	-17.29	<.001
(Sexual experience) Who is the decision maker regarding your contraceptive method.					
	Myself.	-	-	-	-
	My partner.	0.10	0.16	0.61	.54
	By mutual negotiation.	-0.02	0.09	-0.22	.82
	It depends.	0.07	0.15	0.49	.62
	Just use what we can find.	0.51	0.25	2.05	.04
	Others.	0.51	0.55	0.93	.35
(Sexual experience) Availability of contraceptives.					
	Very convenient.	-	-	-	-
	Relatively convenient.	0.08	0.09	0.85	.39
	Relatively inconvenient.	0.11	0.14	0.79	.43
	Very inconvenient.	-0.38	0.28	-1.39	.16
